# Navigating the infodemic: strategies and policies for promoting health literacy and effective communication

**DOI:** 10.3389/fpubh.2023.1324330

**Published:** 2024-01-12

**Authors:** Sheikh Mohd Saleem, Shah Sumaya Jan

**Affiliations:** ^1^Independent Public Health Researcher, Health Section, International NGO, New Delhi, India; ^2^Government Medical College (GMC), Srinagar, Jammu and Kashmir, India

**Keywords:** COVID-19, pandemic, health communication, health literacy, infodemic, misinformation, public health, trust

## Abstract

The COVID-19 pandemic, with its vast impact illustrated by 770 million confirmed cases and 6.9 million deaths as of September 21, 2023, has exposed a critical challenge: the infodemic. Effective communication and health literacy are pivotal in addressing this crisis. This article emphasizes the urgency of combating health misinformation, highlighting its tangible impact on public health and social well-being. Trustworthy sources, especially government agencies and public health officials, played a central role in shaping public behavior. Clear, accurate, and consistent messaging became vital. Health literacy, a fundamental determinant of pandemic response, empowered individuals to understand and act upon health information. Approximately 36% of adults exhibited basic or below-basic health literacy skills, emphasizing its crucial role. Improving health literacy emerged as a strategic imperative, enabling informed choices and proactive health protection. The pandemic underscores the vital role of effective communication and health literacy in combating health misinformation, fostering informed decision-making, and safeguarding public health.

## Introduction

The COVID-19 pandemic has not only brought unprecedented health challenges but has also spotlighted the pivotal role of effective communication and health literacy in navigating the infodemic—a relentless flood of information, ranging from accurate to misleading (1). In this comprehensive article, we embark on a journey to unravel the intricate connection between these two fundamental elements and put forth a series of policy recommendations, firmly rooted in credible sources, to emphasize the pressing need for combating health misinformation during public health crises.

The global COVID-19 pandemic, declared by the World Health Organization (WHO), emerged as an unparalleled global challenge, transcending borders and affecting every facet of society. As of 21st September 2023, the virus has infected 770,778,396 confirmed cases of COVID-19, including 6,958,499 deaths (2), underscoring the urgency of effectively addressing not only the virus itself but also the vast sea of information and misinformation accompanying it.

Amidst the relentless spread of the virus, another epidemic, known as the “infodemic,” (3) swiftly took hold. This digital pandemic, fueled by the proliferation of information channels, inundated the public with a bewildering array of facts, speculations, and falsehoods. Navigating this treacherous landscape became as crucial as adhering to health guidelines, as misinformation and confusion threatened lives and strained healthcare systems.

At the heart of this complex challenge lie two indispensable elements: effective communication (4) and health literacy (5). The former dictates how information is disseminated, understood, and acted upon, while the latter empowers individuals to critically engage with and apply health information to make informed decisions.

As we embark on this exploration, it becomes evident that the fight against health misinformation is not just an abstract concept but a tangible imperative with far-reaching implications for public health social cohesion, and individual well-being. By delving into the intricate relationship between effective communication and health literacy and by aligning our strategies with evidence-based policy recommendations, we endeavor to equip societies with the knowledge and tools needed to conquer the infodemic and emerge from this global challenge stronger and more resilient than before.

## The COVID-19 pandemic: a global crisis

The COVID-19 pandemic, declared by the World Health Organization (WHO) on March 11, 2020, represents one of the most significant global health crises of our time. This declaration had a profound impact on the world as it signaled the gravity of the situation. To truly understand the magnitude of this crisis, it is essential to delve into the details, supported by appropriate data.

- *Global spread of COVID-19:* The COVID-19 virus, caused by the novel coronavirus SARS-CoV-2, exhibited an unprecedented ability to spread rapidly across international borders. As of March 11, 2020, when the pandemic was officially declared, the virus had already infected over 118,000 people in 114 countries, with more than 4,000 fatalities reported worldwide. This data, compiled by the WHO, underscored the virus’s exceptional transmission rate (2).
- *Seismic shift in the battle against COVID-19:* The global pandemic declaration marked a transition from viewing the outbreak as local to recognizing it as a worldwide health emergency. It highlighted the virus’s global presence, emphasizing the need for coordinated international responses.
- *Vulnerabilities in healthcare systems:* The rapid virus transmission exposed global healthcare vulnerabilities. Hospitals worldwide were overwhelmed, especially in countries like Italy, Spain, and the United States, where ICU occupancy rates reached critical levels during the initial wave.
- In Italy, ICU bed occupancy rates exceeded 85%, notably in Lombardy, placing immense pressure on healthcare workers and resources. This emphasized the urgent need for preparedness and surge capacity in healthcare systems (6).
- *Response mechanisms and preparedness:* The pandemic declaration spurred a global reevaluation of response strategies. Governments and health organizations swiftly implemented measures to mitigate the virus’s spread. Countries bolstered testing capabilities, conducting millions of tests weekly to identify and isolate cases promptly. This proactive approach, vital in preventing transmission, was evident in data from the COVID-19 Testing Database (7). Governments enforced lockdowns and social distancing, supported by data modeling studies. Research in journals like Nature Human Behavior showcased the effectiveness of these interventions, emphasizing their role in reducing virus transmission (8).
- *Economic and social impacts:* The pandemic’s repercussions extended beyond healthcare, causing widespread economic contractions. Lockdowns led to job losses and business closures, with a global decline in working hours equivalent to 495 million full-time jobs in Q2 2020, as per the International Labor Organization (9). School closures disrupted education and affected mental well-being. UNICEF data highlighted the struggles faced by families adapting to remote learning and essential service closures (10).

The COVID-19 pandemic’s effects persist, shaping ongoing vaccination efforts and responses to new virus variants. Discussions about global preparedness for future pandemics continue, underscoring the complexity of addressing global health crises.

**Figure 1:**
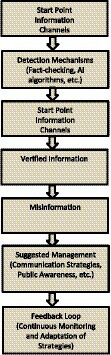
Infodemic information flow chart: detecting, verifying, and managing information during a pandemic.

Figure 1 shows a detailed flow chart illustrating the intricate process of information flow during a pandemic, emphasizing the crucial stages of detection, verification, and strategic management. It begins with the detection of information from various channels, followed by decision points for employing detection mechanisms, leading to either verified information or the potential spread of misinformation. The suggested management phase highlights strategies such as communication and public awareness campaigns. The flow chart emphasizes the cyclical nature of information flow with a feedback loop and continuous adaptation, ultimately leading to the end of the information flow.

## Navigating the emotional landscape: global impacts of COVID-19 misinformation on public well-being

The spread of misinformation, especially during public health emergencies like the COVID-19 pandemic, can have significant emotional impacts on populations across the globe. A growing body of research has explored the psychological effects of misinformation and its ability to evoke strong emotional responses from individuals.

Studies conducted in the United States during the pandemic reveal high levels of anxiety, depression, and anger linked to the consumption of misinformation regarding COVID-19 (11, 12). Exposure to conspiracy theories and false claims induces feelings of powerlessness, confusion, and distrust in authorities, deteriorating mental wellbeing (13). Online misinformation provokes moral outrage, particularly content that politicizes health protocols like masking and vaccination (14).

In Europe, belief in misinformation correlates with increased pandemic-related stress and reduced compliance with preventive behaviors, jeopardizing public safety (15). Experimental studies demonstrate that misinformation elicits anger, leading people to engage in risky actions like protests against pandemic restrictions (16). False claims regarding vaccine side-effects generate anxiety that deters vaccination intent, despite assurances from health authorities (17).

In Africa, misinformation paints the pandemic as a “hoax,” reducing vigilance and protective actions (18). Conspiracy theories linking COVID-19 to 5G technology have spread fear, driven attacks on cell towers, and eroded trust (19). Similarly, misinformation has provoked panicked reactions in Latin America, including gasoline riots in Mexico following false claims that fuel could combat the virus (20).

The negative emotional responses resulting from misinformation are significant barriers to effective pandemic response globally. Public resentment, suspicion, and defiance induced by false claims can jeopardize adherence to protective behaviors (21). Targeted misinformation campaigns aimed at inciting turmoil have been linked to extremist actions like hate crimes against Asian communities during COVID-19 (22).

Addressing the intersection between misinformation and emotions is critical when communicating health information during crises. Strategies should account for the psychology underlying how people react to and spread false claims (23). Promoting media literacy and developing targeted digital interventions can counteract misinformation and mitigate associated public distress (24). Ultimately, evidence-based communication that builds public trust and resilience is essential for navigating health emergencies in an age of rampant misinformation.

## Effective communication: a global necessity

Early in the COVID-19 pandemic, clear and accurate communication emerged as crucial. It became evident that conveying trustworthy information was pivotal for public understanding, cooperation, and adherence to safety guidelines.

- *The role of trustworthy sources:* Amid the COVID-19 crisis, individuals sought trustworthy information. Government agencies and public health officials became key sources of authoritative guidance. Data from the Pew Research Center highlighted the significant reliance on these official channels, reaffirming public trust during a time of uncertainty (25).
- *Official sources as pillars of public trust:* During the pandemic, reliance on public health officials and government agencies was not just preference—it symbolized trust. Trust in authoritative sources became pivotal, shaping public behavior. People adhered to safety measures and guidelines when information came from credible channels. This trust extended to actions like social distancing and mask wearing, as revealed by Pew Research Center data, underlining the impact of trust on public response (25).
- *The implications for effective communication:* Insights from Pew Research Center’s data emphasized effective communication’s pivotal role during the pandemic (25). Government agencies and health officials were entrusted not just with accuracy but also transparency, addressing concerns, and adapting to new data. Additionally, the findings highlighted the necessity of a unified messaging approach. Inconsistencies in messaging eroded trust and cooperation (11). Governments and health organizations thus collaborated closely, ensuring a cohesive, consistent message for the public.
- *The power of trustworthy communication:* Data from Pew Research Center’s early pandemic surveys emphasized the symbiotic link between effective communication and public trust. Credible information shaped public behavior, fostering adherence to safety measures (25).

## Understanding the problem: the role of health literacy

The challenge surpassed effective communication alone, highlighting the pivotal role of health literacy. Proficiency in accessing, comprehending, and applying health information, collectively known as health literacy, became crucial in responding effectively to the crisis.

- *Data from the National Assessment of Adult Literacy (NAAL):* Assessment of Adult Literacy (NAAL) in the United States emphasizes the urgency of addressing health literacy. The assessment revealed a concerning reality: nearly 36% of adults possessed only basic or below-basic health literacy skills (26). This statistic serves as a crucial wake-up call, underscoring the imperative need to recognize and prioritize health literacy as an intrinsic and non-negotiable element within public health efforts (26). In a world inundated with health-related information, health literacy becomes the compass guiding individuals through the complex landscape. It equips them with the tools to decipher intricate medical terminology, critically evaluate health advice, and make informed decisions about their well-being.
- *The vital role of health literacy:* Health literacy proved pivotal during the COVID-19 pandemic, influencing how individuals perceived the virus, evaluated information credibility, and embraced protective measures. Higher health literacy correlated with better adherence to guidelines, informed vaccination decisions, and engagement in behaviors that mitigated the virus’s spread.
- *Elevating health literacy as a public health imperative:* Data from the NAAL highlights a crucial truth: health literacy is not optional but a foundational element of effective public health (26). In navigating the COVID-19 pandemic and future health crises, enhancing health literacy is imperative. It enables individuals to proactively engage in their health, fostering a society that is prepared, informed, and resilient against intricate health challenges.

## The crucial link between health literacy and behavior change

Health literacy is more than comprehending health information; it empowers individuals to make informed choices about their well-being. A WHO report underscores health literacy’s critical role in shaping individual behavior, especially in adopting vital preventive measures like vaccination and strict adherence to safety guidelines. These actions are fundamental in our joint efforts against health misinformation (26).

- *The essence of health literacy:* Health literacy goes beyond comprehension; it involves critically evaluating information, weighing the risks and benefits of health choices, and taking proactive steps for better health outcomes. In a world flooded with health information, those with high health literacy can navigate this complex landscape effectively.
- *The WHO’s insightful report:* The World Health Organization’s report emphasizes the crucial link between health literacy and behavior change, highlighting its significance in combating health misinformation (27). Individuals with higher health literacy not only comprehend health information better but also are also more likely to act upon it. This leads to a greater inclination to adopt preventive measures like vaccination, essential in reducing the risk of contagious diseases.
- *The role of health literacy in the pandemic response:* During the COVID-19 pandemic, the connection between health literacy and behavior change was evident. Individuals with higher health literacy were more likely to adhere to safety measures like mask-wearing, social distancing, and hand hygiene. Additionally, they were more receptive to vaccination as a crucial strategy to combat the virus (28).
- *Empowering the individual:* Health literacy empowers individuals to navigate the complexities of health information, enabling them to make informed decisions aligned with their well-being. In a world inundated with health misinformation, health literacy acts as a protective shield, offering the knowledge and confidence needed to discern between accurate and inaccurate information.
- *The vital nexus:* The crucial connection between health literacy and behavior change stands as a cornerstone in our battle against health misinformation. It emphasizes that health literacy is not merely a passive trait but an active force propelling individuals toward informed choices and decisive actions for their health. Acknowledging and promoting health literacy, as emphasized by the WHO, is not just a public health necessity; it is a fundamental strategy to empower individuals and strengthen our collective defenses against health misinformation in our complex, interconnected world (27).

## Behavioral models: shaping perceptions and actions

Understanding how individuals perceive and respond to health-related information is a multifaceted endeavor, often informed by various behavioral models. These models serve as valuable frameworks for comprehending the intricate interplay between knowledge, perception, and behavior in the context of health. One such influential model is the Health Belief Model, widely utilized in public health, which provides key insights into the factors influencing individuals’ health-related decisions and actions (29).

### The health belief model

A Foundation in Public Health: The Health Belief Model (HBM) postulates that an individual’s perception of the threat posed by an illness, coupled with their assessment of the benefits and barriers associated with preventive actions significantly shape their health-related behavior (29). This model acknowledges that individuals weigh the perceived risks of an illness against the perceived benefits of taking preventive measures when making health-related decisions.

## The complexity of behavioral change

Changing behavior is a complex and multifaceted endeavor that demands a nuanced approach, characterized by patience, time, and the implementation of comprehensive strategies. A study published in the Journal of Epidemiology and Community Health sheds light on the intricate nature of behavior change, emphasizing that it is an incremental process that necessitates continuous education and reinforcement. In this context, health promotion activities must extend beyond the mere dissemination of information and embrace practical approaches that seamlessly integrate health-promoting behaviors into individuals’ daily lives (30).

- *The nature of behavior change:* Behavior change is a journey rather than a momentary event. It involves altering ingrained habits and adopting new, health-enhancing practices. This process can be arduous and is often marked by setbacks and relapses. Understanding the gradual nature of behavior change is crucial for designing effective interventions and initiatives.
  - *Practical approaches for integration:* Incorporating health-promoting behaviors into daily life is paramount for sustainable change. It involves more than simply imparting knowledge; it requires creating an environment that fosters and supports these behaviors (31).

## Setting-based approaches for behavioral change

Educational institutions and community settings have a profound influence on individuals, particularly during their formative years. It is in these environments that habits and behaviors, including those related to health and hygiene, are often instilled. Data from the Global Initiative for Children’s Surgery (GICS) provide compelling evidence of the transformative power of teaching health and hygiene practices to children (32). Early education in essential practices such as hand hygiene, cough etiquette, and physical distancing not only has immediate benefits but also lays the foundation for lifelong health behaviors (33).

- *The impact of early education:* Education is a potent tool for empowering individuals with the knowledge and skills needed to make informed decisions about their health. When imparted from an early age, this education can have a profound and lasting impact.
- *Fostering lifelong health behaviors*

- *Hand hygiene:* Teaching children the importance of regular handwashing with soap and water instills a habit that can significantly reduce the risk of infections. Moreover, this practice often becomes second nature and persists into adulthood, contributing to better overall health.
- *Cough etiquette:* Educating children about covering their mouths and noses when coughing or sneezing not only prevents the spread of germs but also promotes a sense of responsibility for the well-being of others. These lessons in empathy and hygiene can have far-reaching effects.
- *Physical distancing:* Early education about the benefits of maintaining physical distance in crowded settings can promote a sense of personal space and awareness of the importance of reducing disease transmission. This awareness can extend into adulthood, influencing behavior in various social contexts.

c *The role of educational institutions*: Educational institutions, including schools and early childhood education centers, serve as fertile ground for imparting health education. Incorporating health and hygiene education into the curriculum not only equips students with essential life skills but also establishes a culture of health-consciousness within the institution.
d *Community settings as reinforcement*: Community settings complement the efforts of educational institutions by reinforcing health messages and practices. Local community organizations, clubs, and programs can provide additional opportunities for children to engage with health education and put it into practice in real-life scenarios.

## National health programs: a vehicle for behavioral change

National health programs wield significant influence in shaping the health landscape of a country. These programs not only provide essential healthcare services but also serve as platforms for targeted behavioral change interventions among adults.

- *The significance of national health programs:* National health programs are instrumental in addressing a wide range of health concerns, from communicable diseases to non-communicable conditions. These programs have the infrastructure and reach to engage with diverse segments of the population, making them ideal vehicles for implementing behavioral change interventions.
- *India’s national tuberculosis elimination program (NTEP):* The NTEP in India serves as a noteworthy example of how national health programs can promote behavioral change. Tuberculosis (TB) remains a significant public health challenge in India, and the NTEP has been at the forefront of efforts to combat the disease (34).
- *Educating patients about preventive measures:* One of the key components of the NTEP’s strategy involves educating TB patients about preventive measures. Patients undergoing TB treatment are not only provided with medical care but are also educated about practices such as respiratory hygiene, cough etiquette, and the importance of completing their prescribed treatment regimen (34).
- *The role of health literacy:* Central to the success of such interventions is the enhancement of health literacy among patients. Patients need to understand not only the nature of their condition but also the preventive measures they can take to protect themselves and others from infection. Health literacy equips individuals with the knowledge and skills to make informed decisions about their health (34).
- *Extending the model to other health concerns*: The success of the NTEP’s efforts in educating TB patients about preventive measures serves as a model that can be applied to other health concerns. Similar initiatives can be implemented for diseases such as HIV/AIDS, vector-borne illnesses, and non-communicable diseases like diabetes and hypertension.
- *The broader impact*: Behavioral change interventions within national health programs have a ripple effect. When individuals are educated about preventive measures and adopt healthier behaviors, it not only benefits them individually but also contributes to community-wide health improvements. Reduced disease transmission, lower healthcare costs, and improved overall well-being are some of the far-reaching outcomes.

## Public awareness and health-promoting behaviors

- Public spaces, ranging from restaurants to party venues, serve as significant arenas for promoting health behaviors. Within this domain, the hospitality sector plays a pivotal role in enforcing and championing health-promoting practices. A study published in the International Journal of Hospitality Management offers compelling evidence of how the hospitality sector can be an effective advocate for practices such as handwashing and physical distancing, showcasing the sector’s potential to contribute to public health in meaningful ways (35).
- *The influence of public spaces on health behaviors:* Public spaces are where people congregate and interact, making them influential settings for shaping health behaviors. These spaces offer a unique opportunity to instill and reinforce practices that contribute to individual and community health. The choices made within these settings can have a significant impact on the well-being of patrons and the wider population.
- *The hospitality sector as a driver of health behaviors:* The hospitality sector, comprising restaurants, party venues, and related establishments, holds a particular position of influence within public spaces. These settings can actively promote and enforce health-promoting practices, making them more than just venues for leisure and entertainment (35).

## The need for a comprehensive approach

In the face of health crises, swift technological solutions such as vaccines and diagnostic tests are undoubtedly essential tools. However, a report from the World Health Organization (WHO) emphasizes the critical need for comprehensive, long-term strategies to address the broader context of public health (36). Health promotion activities, driven by health literacy, require time, patience, and collaboration across sectors. This comprehensive approach recognizes that while immediate responses are vital, sustainable solutions demand a multifaceted and enduring commitment.

- *The role of swift technological solutions:* Swift technological solutions like vaccines and diagnostic tests play an indispensable role in controlling the spread of diseases during acute situations (37). They provide immediate relief by identifying cases, isolating infected individuals, and immunizing populations. These tools are essential in preventing widespread outbreaks and saving lives in the short term.
- *The imperative for comprehensive, long-term strategies:* While swift technological solutions are crucial for containing acute crises, they are not standalone remedies. The WHO report highlights that focusing solely on immediate responses without addressing the underlying factors contributing to public health challenges is inadequate. Instead, it calls for comprehensive, long-term strategies that address the broader determinants of health (37).
- *The role of health promotion activities:* Health promotion activities are integral components of these comprehensive strategies. These initiatives aim to empower individuals and communities with the knowledge, skills, and resources they need to make informed decisions about their health. Health promotion encompasses a wide range of activities, from educating the public about disease prevention to promoting healthy behaviors and lifestyles (36, 37).
- *The importance of health literacy:* At the heart of effective health promotion activities lies health literacy. Health literacy is the ability of individuals to access, understand, evaluate, and apply health information to make informed decisions about their health. It is a fundamental aspect of enabling people to take control of their well-being and participate actively in disease prevention and health promotion (1).
- *Multisectoral collaboration:* Collaboration across sectors is another crucial aspect of comprehensive, long-term strategies. Public health is influenced by a multitude of factors, including education, housing, employment, and socioeconomic status. Effective strategies require coordination among various sectors to address these determinants of health comprehensively.

## Multi-sectoral collaboration: the missing link

Effectively controlling infectious diseases demands more than isolated efforts, it necessitates a collaborative approach that harnesses the collective expertise of professionals across various fields.

- *The complexity of infectious disease control:* Infectious diseases, whether emerging or established, often present multifaceted challenges that transcend the boundaries of a single discipline. Addressing these challenges effectively requires an integerated and interdisciplinary approach. The CDC’s data reveals that outbreaks frequently demand the convergence of expertise from diverse fields, each contributing unique insights and strategies (38).
- *Collaborative synergy in action:* Collaboration among experts from these diverse fields can yield powerful results (39).

### Policy recommendations for improvement

Policy recommendations from various sources stress the urgency of addressing health literacy:

- *Incorporate health literacy into education:* Integrating health literacy into school curricula can equip future generations with the skills needed to critically assess health information. This proactive approach fosters a culture of health literacy from an early age.
- *Promote digital health literacy:* Given the digital landscape’s significance, promoting digital health literacy is crucial. People must be adept at navigating online health resources and recognizing trustworthy sources.
- *Tailored communication:* Policymakers should prioritize tailoring health communication to diverse populations, accounting for varying levels of health literacy. Communication materials should be clear, accessible, and culturally sensitive.
- *Health literacy assessment:* Conducting regular assessments of health literacy levels within communities can inform targeted interventions and ensure that resources are directed where they are most needed.
- *Public-private partnerships:* Collaboration between public health agencies, educational institutions, healthcare providers, and the private sector can amplify efforts to improve health literacy.
- *Establish interdisciplinary task forces:* Governments should establish interdisciplinary task forces during public health crises. These task forces should bring together experts from various fields to develop comprehensive strategies for disease control. Clear lines of communication and shared responsibilities can enhance coordination and response effectiveness.
- *Establish health and safety standards:* Policymakers should implement and enforce health and safety standards within public establishments. This includes requirements for hand hygiene, sanitation, and physical distancing. Incentives and recognition programs can encourage compliance among businesses, ensuring the safety of patrons.

## Conclusion

Building a health-literate society: addressing the challenge of health misinformation necessitates a dual approach that combines effective communication with a focus on health literacy. The lessons learned from the COVID-19 pandemic call upon policymakers to prioritize these strategies as part of a broader public health agenda. By building a health-literate society that can critically engage with health information, policymakers can effectively combat misinformation, promote informed decision-making, and ultimately improve the health and well-being of their populations.

## Data availability statement

The original contributions presented in the study are included in the article/supplementary material, further inquiries can be directed to the corresponding author.

## Author contributions

SS: Conceptualization, Data curation, Formal analysis, Investigation, Methodology, Supervision, Validation, Visualization, Writing – original draft, Writing – review & editing. SJ: Investigation, Methodology, Project administration, Validation, Visualization, Writing – original draft.
